# The Role of Interface Geometry and Appendages on the Mesoscale Mechanics of the Skin

**DOI:** 10.21203/rs.3.rs-3182434/v1

**Published:** 2023-07-24

**Authors:** Omar Moreno Flores, Manuel K. Rausch, Adrian B. Tepole

**Affiliations:** 1 School of Mechanical Eng., Purdue University, West Lafayette, USA; 2 Department of Aerospace Engineering and Engineering Mechanics, The University of Texas at Austin, Austin, USA; 3 Weldon School of Biomedical Eng., Purdue University, West Lafayette, USA

**Keywords:** Mechanosensation, Skin biomechanics, Representative volume element, Multiscale tissue mechanics, Interface mechanics, Hair follicle biomechanics

## Abstract

The skin is the largest organ in the human body and serves various functions, including mechanical protection and mechanosensation. Yet, even though skin’s biomechanics are attributed to two main layers - epidermis and dermis-computational models have often treated this tissue as a thin homogeneous material or, when considering multiple layers, have ignored the most prominent heterogeneities of skin seen at the mesoscale. Here we create finite element models of representative volume elements (RVEs) of skin, including the three-dimensional variation of the interface between the epidermis and dermis as well as considering the presence of hair follicles. The sinusoidal interface, which approximates the anatomical features known as Rete ridges, does not affect the homogenized mechanical response of the RVE but contributes to stress concentration, particularly at the valleys of the Rete ridges. The stress profile is three-dimensional due to the skin’s anisotropy, leading to high-stress bands connecting the valleys of the Rete ridges through one type of saddle point. The peaks of the Rete ridges and the other class of saddle points of the sinusoidal surface form a second set of low-stress bands under equi-biaxial loading. Another prominent feature of the heterogeneous stress pattern is a switch in the stress jump across the interface, which becomes lower with respect to the flat interface at increasing deformations. These features are seen in both tension and shear loading. The RVE with the hair follicle showed strains concentrating at the epidermis adjacent to the hair follicle, the epithelial tissue surrounding the hair right below the epidermis, and the bulb or base region of the hair follicle. The regions of strain concentration near the hair follicle in equi-biaxial and shear loading align with the presence of distinct mechanoreceptors in the skin, except for the bulb or base region. This study highlights the importance of skin heterogeneities, particularly its potential mechanophysiological role in the sense of touch and the prevention of skin delamination.

## Introduction

Understanding and correctly simulating human skin’s mechanical properties is crucial to improving a wide range of medical applications and gaining fundamental knowledge of skin mechanophysiology. For example, skin’s mechanical behavior can influence pressure ulcer development [5], skin growth in tissue expansion [20], drug delivery with auto-injector devices [43], and interaction with prostheses [31]. The mechanical behavior of skin has been characterized primarily as a whole tissue (on the scale of mm to cm) [34, 29]. However, skin is a multi-layered organ with heterogeneous composition and microstructure (on the scale of *μ*m to mm). The individual layer properties and their interaction contribute to the unique mechanics of skin at the macroscale [52]. However, even though recent efforts have acknowledged the effect of considering multiple layers for an accurate understanding of skin’s mechanical behavior, existing models still ignore the intricate interface geometry between layers and the presence of skin appendages [40, 54, 12].

The epidermis is the top layer of the skin. It is an epithelial layer and is, thus, mostly made from cells called keratinocytes. The epidermis plays a role in the overall skin mechanical properties primarily at small tensile deformations, during compression, and in the contact mechanics against other surfaces [14, 1]. It is also an essential component of the tactile sense [55]. Several nerve endings and appendages are embedded in the epidermis, such as Merkel cells and Meissners’ corpuscles, which are known for their role in the sense of touch [55]. The dermis is the middle layer of the skin. It is the major load-bearing layer at larger tensile stretches because of its collagen network [34, 32, 38]. The hypodermis is the bottom layer. It is a soft tissue that connects the dermis to the underlying muscle tissue. The hypodermis or subcutaneous tissue is important during compression and for its role in transport during subcutaneous drug delivery [44]. This study focuses on the epidermis and dermis layers and their role in skin biomechanics under tension and shear. In addition to each layer’s unique composition and structure, we specifically model the wavy surface between the dermis and epidermis. That is, we model the array of sinusoidal-like peaks and valleys at the dermis-epidermis interface, so-called Rete ridges [42]. Skin properties do not only differ through thickness but also with anatomical location. Therein, we can differentiate between two main types of skin, glabrous skin, and hairy skin [33]. As the name indicates, hairy skin contains hair follicles, while glabrous skin lacks hair. Hairy skin varies further in the geometry of its hair follicles and their density, e.g., the scalp versus the rest of the body [49, 7, 46]. The glabrous skin is found in the palms of our hands and feet. Here we create a detailed three-dimensional (3D) skin model including a general sine wave interface to capture the effect of the Rete ridges on the epidermis-dermis interface. Our model also includes hair follicles to elucidate their possible role in tactile sensation and overall skin properties.

Owing to their different composition, different skin layers have distinct mechanical behavior. The epidermis is largely comprised of keratinocyte cells. Owing to its small thickness, its mechanics have been measured primarily via indentation [13, 24]. To this end, inverse finite element models have been created to infer individual layer properties from this complex loading mode. From these experiments, we know that the epidermis behaves isotropically and, while moderately nonlinear, can be modeled with hyperelastic potentials such as the Ogden and neo-Hookean forms. In contrast to the epidermis, the dermis is comprised largely of collagen and elastin [23]. At low stretches, it is very soft, and mechanical properties can be attributed mostly to elastin [27]. As the tissue is stretched, collagen fibers uncrimp and exhibit an exponential-like strain stiffening [8]. Additionally, the dermis tissue is anisotropic, with its axes of symmetry depending on where the tissue was excised from as best described in the so-called Langer Lines[2]. Models of dermal biomechanics have been calibrated against either tensile test data of whole tissue or individual layers, or against inverse finite element models based on indentation and suction measurements [45, 35]. The most popular hyperelastic potentials to describe dermal mechanics have been the Holzapfel, Ogden, and Gasser (HGO) potentials [34].

Using the above information about the different mechanical behavior of individual layers, interface geometry, and hair follicle geometry, we create a representative mesoscale volume element (RVE) of skin on the order of 1mm^3^ with features on the order of 100*μ*m. We test this model under tension and shear configurations. The study is thus designed to shed light on how the strain and stress distribution in the skin are affected by the interface and appendage geometries. This study thus fills the gap in our understanding of how heterogeneities in skin tissue impact its mechanical behavior at larger scales and conversely, how large-scale deformations translate into stress and strain concentrations that might be the key to understanding skin mechanosensing and mechanobiology.

## Methods

To study the mechanics of skin at the mesoscale, we created a representative volume element (RVE) that features an epidermis and dermis layer, a sinusoidal interface between both layers, and also includes hair follicles. Further, we deform this model under both stretch and shear to elucidate the role of anisotropy, material properties, and interface parameters.

### Finite element model

**Fig. 1:**
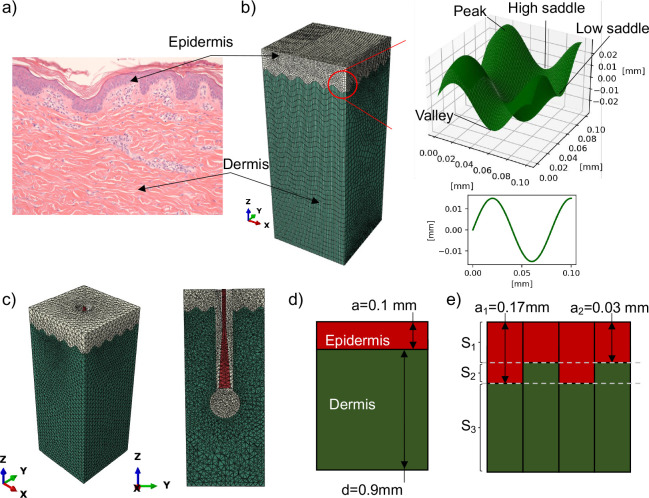
Representative volume elements (RVE) of skin. a) Histology image from porcine skin, with epidermis at the top and dermis at the bottom. b) Histology measurements of epidermis thickness were used to create a finite element model of a skin RVE with a sinusoidal interface between the epidermis and dermis. c) RVE with a hair follicle. d) Flat interface model used for the analytical results based on the rules of mixtures. e) Modified interface used for the semi-analytical results.

The RVE model consists of the two skin layers that majorly contribute to skin’s mechanical and mechanobiological response under tension and shear: the epidermis and the dermis (Fig. 1a). The epidermis was assigned an average thickness of 0.1 mm. The dermis was modeled as 0.9 mm thick on average. The width and length of the model was set to 0.4002 mm in order to achieve periodicity of the RVE. The sine wave interface was modeled with the parametric equation

(1) x=ty=Asin(Bt)

where A is the amplitude and B the period. For the baseline model, A=0.015mm,B=78.51/mm, determined by measuring the vertical and horizontal distance between peaks and valleys of Rete ridges on skin histology images in ImageJ (Fig. 1b, see also Supplemental Material). Several variations of this model were created. One model was assigned equal thickness to epidermis and dermis layers in order to investigate the contribution of the different materials to the homogenized response while ignoring the role of volume fraction of each layer. A set of five models were generated by changing the A,B values of the sine interface, described below. The last finite element model was created with a hair follicle and surrounding epithelium at the center of the standard skin model containing the epidermis and dermis. The hair follicle geometry was based on average anatomical measures reported in [46] as well as using a histology images of a hair follicle in [49](Fig. 1c, see also Supplemental Material).

To compare the finite element model against analytical approximations, we considered an ideal model with a flat interface solved using the rule of mixtures (Fig. 1d) and a semi-analytical model solved by considering a composite with three different arrangement of material strips loaded in parallel. The three strips were either homogeneously made out of epidermis or dermis material, or an alternating pattern of epidermis and dermis (Fig. 1e). RVE meshes of the analytical models were also built for verification of the finite element simulation setup.

All of the simulations were conducted using Abaqus Standard. For the baseline model (Fig. 1a), we identified a mesh of 195704 hexahedral hybrid elements C3D8H and tetrahedral elements C3D6H as converged. For the hair follicle RVE we found that 202074 C3D10H and C3D10 elements were necessary for convergence. To correctly approximate the homogenized material response, periodic boundary conditions (PBC) were imposed as linear constraints between corresponding nodes on opposite faces. The only exception were the hair follicle simulations as the mesh did not allow us to identify a bijective map between nodes on opposite faces. For the hair follicle geometry, Dirichlet boundary conditions were imposed on the lateral faces of the RVE.

The baseline RVE was deformed in strip-x $\left(\lambda_{x}\in \right.\;\left.[1,\;1.5],\lambda_{y}=1\right)$, strip-y $\left(\lambda_{y}\;:\in [1,\;1.5],\lambda_{x}=1\right)$, and equi-biaxial deformation ($\lambda_{x}\;:\lambda_{y}=1\;:1$ up to $\lambda_{x}=1.5$). Shear simulations were also performed on this model by displacing the top surface in the x-direction by $u_{sx}=0.2\;mm$. The RVE with equal epidermis-dermis thickness and the RVEs with varying amplitude and period of the sine interface were subjected only to equi-biaxial deformation. The hair follicle model was subjected to both equi-biaxial and shear deformations.

To obtain the homogenized properties from the RVEs, reaction forces on the boundary faces were integrated and divided by the corresponding area. The homogenized stresses for each deformation are denoted as $\sigma^{h}$. Additionally, we were interested in the absolute value of the stress jump across the epidermis-dermis interface denoted as 〚σ〛.

### Analytical model

Two simple models were used to estimate the homogenized properties of the skin tissue. The first was the rule of mixtures, which simply considers the additive split of the strain energy (or the stress tensor) based on the initial volume fraction of each material

(2) $$W^{h}(F)=\phi_{E}W_{E}(F)+\phi_{D}W_{D}(F)$$

were $W^{h}$ is the homogenized strain energy, while $W_{E}$, $W_{D}$ are the strain energies describing the material response of epidermis and dermis, respectively. This approach assumes that both domains are subject to the same deformation gradient F. The homogenized response is then a convex interpolation between the response of either material in terms of the volume fraction of epidermis and dermis $\phi_{E}$, $\phi_{D}$, which satisfy $\phi_{E}+\phi_{D}=1$. For example, for the case of strip-x biaxial deformation and assuming incompressible behavior (J=detF=1), $\lambda_{x}=\lambda \in [1,\;1.5]$, $\lambda_{y}=1$, $\lambda_{z}=1/\lambda$, and the stress of the mixture in the x direction follows as

(3) $$\sigma^{h}(\lambda )=\phi_{E}\lambda \frac{\partial W_{E}(\lambda )}{\partial \lambda }+\phi_{D}\lambda \frac{\partial W_{D}(\lambda )}{\partial \lambda }+p,$$

where the Lagrange multiplier p can be determined via the plane stress condition. The specific material models for epidermis and dermis are detailed later.

For the second analytical approach we considered the three strips in Fig. 1e. Under strip-x biaxial loading the stress in the x-direction in the top and bottom strips, $S_{1}$ and $S_{3}$, is that of a uniform material made out of either dermis or epidermis,

(4) $$\sigma_{S1}=\sigma_{E}(\lambda )=\lambda \frac{\partial W_{E}(\lambda )}{\partial \lambda }+p_{E}$$

(5) $$\sigma_{S3}=\sigma_{D}(\lambda )=\lambda \frac{\partial W_{D}(\lambda )}{\partial \lambda }+p_{D},$$

where $p_{E}$ and $p_{D}$ are Lagrange multipliers to enforce incompressible behavior of either dermis or epidermis layers. The middle strip $S_{2}$ can be seen as springs in series made out of either dermis or epidermis materials. In accordance with Newton’s third law, the stress has to be fully transferred across the dermis and epidermis domains such that

(6) $$\sigma_{S2}=\sigma_{E,S2}=\sigma_{D,S2}\;=\lambda_{E,S2}\frac{\partial W_{E}\left(\lambda_{E,S2}\right)}{\partial \lambda_{E,S2}}+p_{E,S2}\;\;=\lambda_{D,S2}\frac{\partial W_{D}\left(\lambda_{D,S2}\right)}{\partial \lambda_{D,S2}}+p_{D,S2}.$$

Eq. (6) has to be solved for $\lambda_{E,S2}$, $\lambda_{D,S2}$ with the additional constraint that $\left(\lambda_{E,S2}+\lambda_{D,S2}\right)/2=\lambda$. The solution of these two equations does not necessarily have a simple closed-form solution, depending on the material models $W_{E}$, $W_{D}$. In practice we solve the system of equations numerically with Newton-Raphson iterations. The homogenized response becomes

(7) $$\sigma^{h}\left(\lambda \right)=a_{1}\sigma_{S1}+\left(a_{2}-a_{1}\right)\sigma_{S2}+\left(1-a_{1}\right)\sigma_{S3}.$$

### Constitutive equations

The epidermis and epithelium surrounding the hair follicle were modelled using the nearly incompressible Ogden material model [15],

(8) $$W\left({\bar{\lambda }}_{1},{\bar{\lambda }}_{2},{\bar{\lambda }}_{3},J\right)=\frac{\mu_{E}}{\alpha^{2}}\left({\bar{\lambda }}_{1}^{\alpha }+{\bar{\lambda }}_{2}^{\alpha }+{\bar{\lambda }}_{3}^{\alpha }-3\right)+D_{E}^{-1}{\left(J-1\right)}^{2},$$

where ${\overset{‾}{\lambda }}_{i}=J^{-1/3}\lambda_{i}$ are the isochoric principal stretches, $\lambda_{i}$ being the principal stretches of the deformation, and $J=\lambda_{1}\lambda_{2}\lambda_{3}$ the volume change. The Ogden model is parameterized by α, $\mu_{E}$, $D_{E}$. Note that for the analytical calculations the incompressibility assumption was enforced exactly while nearly incompressible behavior was used in the finite element simulations.

The dermis was modelled using the Gasser–Ogden–Holzapfel (GOH) model and it was assumed nearly incompressible [36]. The strain energy function for the nearly incompressible GOH model reads

(9) $$W=\frac{\mu_{D}}{2}\left(I_{1}-3\right)+\frac{k_{1}}{2k_{2}}(e^{k_{2}{\left[\kappa I_{1}+(1-3\kappa )I_{4}-1\right]}^{2}}-1)\;\;+D_{D}^{-1}(J-1{)}^{2},$$

where $I_{1}=tr(C)$, $I_{4}=a_{0}\cdot C\cdot a_{0}$ are invariants of the Cauchy Green deformation tensor, and $\mu_{D}$, $k_{1}$, $k_{2}$, κ, $D_{D}$ are material parameters. We directly set $D_{D}=0$ to denote the incompressible response in Abaqus. The first invariant is standard in hyperelastic material models [cite], while the other invariant depends on the choice of an anisotropy direction $a_{0}$. In our simulations we set $a_{0}$ to be aligned with the x-axis.

The hair follicle was modeled using the compressible isotropic neo-Hookean model [18],

(10) $$W\left(\lambda_{1},\lambda_{2},\lambda_{3},J\right)=\mu_{H}\left(\lambda_{1}^{2}+\lambda_{2}^{2}+\lambda_{3}^{2}-3\right)+D_{H}^{-1}{\left(J-1\right)}^{2},$$

parameterized by $\mu_{H}$, $D_{H}$. The material parameters used in Abaqus for the material models are shown in Tables 1–3.

## Results

### Homogenized properties are dominated by the dermis and are independent of the interface geometry

**Fig. 2:**
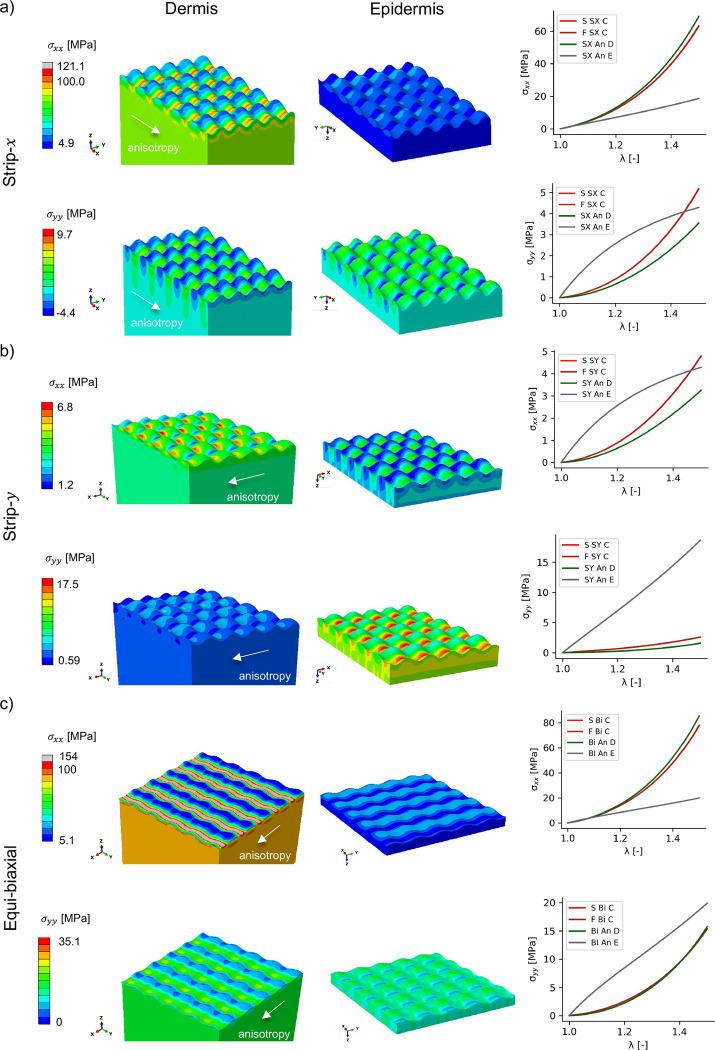
RVE simulations show stress concentrations, but the homogenized behavior is independent of the sinusoidal interface. The RVE was simulated under strip-x (a), strip-y (b), and qui-biaxial loading (c) to obtain homogenized stresses $\sigma_{xx}^{h}$ and $\sigma_{yy}^{h}$. The homogenized response was obtained from RVEs with flat (F) or sinusoidal (S) interface and compared against the analytical (An) stress curves of either bulk material, dermis (D), or epidermis (E). The homogenized response is independent of the interface geometry, but the simulations show how regions of higher epidermis content (valleys of the sinusoidal wave from the perspective of the dermis) induce larger $\sigma_{xx}$ stresses in the dermis, which is the stiffer material in that loading direction due to its anisotropy.

In Fig. 2, the homogenized stresses $\sigma_{xx}^{h}$ and $\sigma_{yy}^{h}$ of the RVE tested in strip-x, strip-y and equi-biaxial deformation are between the curves corresponding to the analytical models of either dermis or epidermis bulk materials. Since the dermis is described with the GOH strain energy, the analytical response of this material is highly nonlinear, with the classical J-shaped response at increasing stretches. On the other hand, the epidermis, modeled with the Ogden potential, shows a more linear response. The RVE, consisting mostly of dermis material, shows homogenized response closer to the dermis than to the epidermis analytical models.

Because the dermis is modeled as an anisotropic solid in accordance to the parameters in [cite], when tested in strip-x biaxial loading, the dermis stress in the direction of anisotropy is much greater compared to the epidermis stress $\sigma_{xx}^{D}>\sigma_{xx}^{h}>\sigma_{xx}^{E}$, but the dermis stress in the y-direction is actually smaller than the epidermis counterpart $\sigma_{yy}^{D}<\sigma_{yy}^{h}<\sigma_{yy}^{E}$. In strip-y biaxial loading, which does not stretch the fiber family of the dermis, the epidermis stresses are greater than dermis stresses in both directions, $\sigma_{xx}^{D}$, $\sigma_{yy}^{D}<\sigma_{xx}^{h}$, $\sigma_{yy}^{h}<\sigma_{xx}^{E}$, $\sigma_{yy}^{E}$. In equi-biaxial loading, similar trends to the strip-x case were seen. These results show the importance of multilayer models depending on the type of loading applied to the skin.

The homogenized response from the RVE with the sinusoidal interface, a finite element model with a flat interface, and the rule of mixtures, all show the same homogenized response (Fig. 2). Therefore, the sinusoidal interface does not contribute to the homogenized biaxial mechanics of skin. On the other hand, there are significant stress variations along the sinusoidal interface. For the dermis, stresses in the direction of anisotropy $\sigma_{xx}$ are greater in the valleys of the sinusoidal interface (where the epidermis is thicker) compared to the peaks (where the epidermis is thinner). Furthermore, even though the sinusoidal interface has the same periodicity in x and y, for $\sigma_{xx}$ the higher stresses are not just the valleys of the interface, rather, there are bands of high stress which are perpendicular to the direction of anisotropy (see for example $\sigma_{xx}$ in Fig. 2e). For the regions of low stress in the dermis, the $\sigma_{xx}$ values are close to the values observed in the epidermis regions right across the interface. For the epidermis, regions of high and low stress depend on the type of loading. For strip-x and equi-biaxial loading, regions of high stress are seen in regions of higher epidermis thickness (which are the valleys from the perspective of the dermis). For the strip-y loading the stress concentration in the epidermis is reversed, with the regions of smaller epidermis thickness having the larger stresses. This is explained by the overall trends mentioned before, that the dermis is much stiffer in the direction of anisotropy compared to the epidermis, but the epidermis being stiffer when loaded in the strip-y mode.

### The sinusoidal interface reduces the average stress jump along the interface

**Fig. 3:**
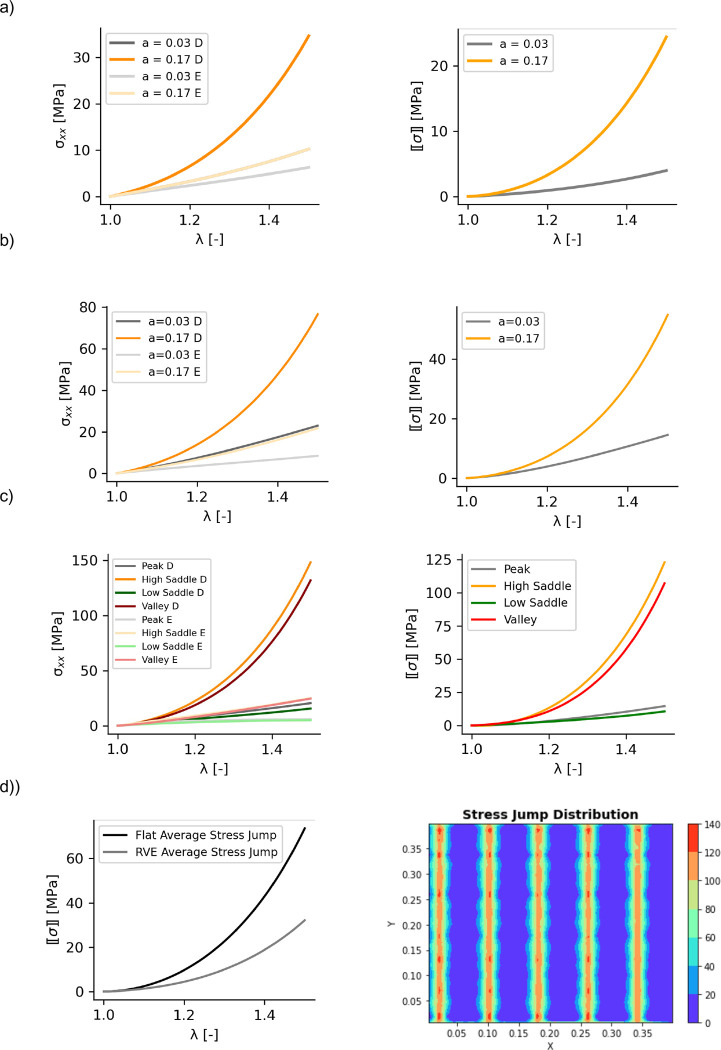
Stress jumps are smaller in the sinusoidal interface compared to the flat interface between epidermis and dermis. a) Semi-analytical model from Fig. 1e loaded in strip-x. Stresses are reported for the different locations along the Rete ridges, i.e. different epidermis amplitude (a=0.03mm or a=0.17mm) for either the dermis side (D) or the epidermis side (E). (b) Results from a finite element model with the same geometry as Fig. 1e shows the same results in strip-x loading, providing confidence in the semi-analytical approach. c) Finite element simulation for the RVE with the sinusoidal interface shows the variation in stress for all locations of interest, as well as the stress jump at each of those locations. d) The absolute value of the stress jump is on average higher on the flat interface model compared to the sinusoidal interface RVE model.

Considering the strip-x biaxial loading, the simplified model with the three strips loaded in parallel, illustrated in Fig. 1e, was solved. Figure 3a left shows the results for this analytical model. There are three strips but four stress values as indicated in Fig. 3a, corresponding to eqs. (5) and (6). The corresponding stress jumps from the semi-analytical model are shown in Fig. 3a, on the right. To verify these results, a finite element model with the same geometry as illustrated in Fig. 1e was created and similar results were obtained in strip-x loading, shown in Fig. 3b. From this analysis we observed that the stress jump increased with stretch and was greater in the *valleys* of the sinusoidal interface (greater amplitude of epidermis material =0.17mm) compared to the *peaks* of the interface (for which the epidermis amplitude was a=0.03mm).

The same trends were also observed in the finite element model with the sinusoidal interface, shown in Fig. 3c. Under strip-x biaxial loading, the interface location corresponding to valleys in the dermis had a greater stress jump compared to the interface at dermis peaks. In the model with the full sinusoidal interface, in addition to peaks and valleys, two other locations of interest were identified as *high saddle* and *low saddle* (see also Fig. 1). The high saddle followed the trends of the valley, whereas the stress jump across the low saddle resembled the response at the dermis peaks. We reiterate that peaks from the dermis point of view are regions with lower epidermis thickness, and valleys in the dermis are those with greater epidermis thickness.

The most interesting result was the comparison of the stress jump between the model with the sinusoidal interface and the flat interface model. The sinusoidal interface showed a lower average stress jump than the model with the flat interface. The contour of the stress jump in Fig. 3d shows that the stress jump is concentrated along strips that run through the entire width of the model, which is why we see similar trends between valleys and low saddles and between peaks and high saddles. These strips of high stress jumps are oriented orthogonal to the direction of anisotropy which, for this example, coincided with the direction of loading in the x-direction. These results suggest that even though the sinusoidal interface increased stress concentration in both dermis and epidermis and did not affect the homogenized response, the reduction of the stress jump can have mechanophysiological advantages, e.g. potential impact on delamination properties.

### There is a transition in the load carrying layers as a function of deformation

Because the epidermis material is more linear compared to the dermis response, we noticed in the simulations of Fig. 2 that the epidermis stresses could actually dominate the homogenized response at low stretches for which the dermis response is soft. In Fig. 2, the more prominent contribution of the epidermis to the homogenized response was mostly observed in the stresses orthogonal to the direction of anisotropy. However, because of the J-shaped stress-stretch curve of the GOH model, we anticipated that even in the direction of anisotropy we would see a dominant role of the epidermis depending on the nonlinearity of the GOH model. We therefore decided to employ an RVE with 50% volume of epidermis and dermis and vary the parameter $k_{1}$ related to collagen stiffness in the dermis.

**Fig. 4:**
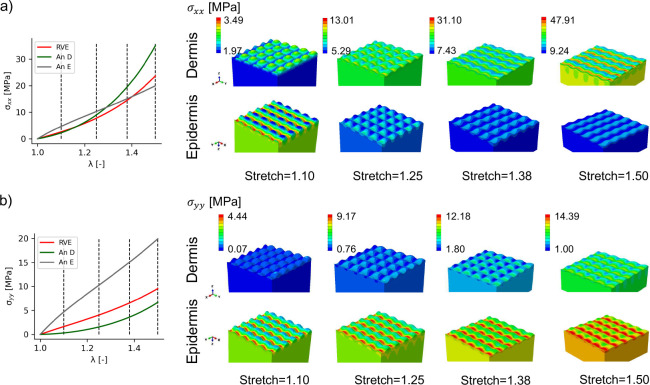
Stress transition at the interface depends on the nonlinearity of the dermis and epidermis properties. Loaded in equi-biaxial deformation, a RVE made out of 50% dermis and 50% epidermis material and with low collagen stiffness $k_{1}=10$ MPa shows that there can be a complete switch in the stress concentration pattern at the interface because the epidermis can be stiffer at low deformations compared to the dermis. The analytical response of either dermis or epidermis (An-D and An-E respectively) are plotted together with the homogenized RVE response for $\sigma_{xx}$ (a) and $\sigma_{yy}$ (b). Due to the high nonlinearity of the dermis model, eventually this material dominates the homogenized response and shows band of high stress for regions with high epidermis content (valleys of the sinousoidal wave from the perspective of the dermis). The transition is more evident in $\sigma_{xx}$ compared to $\sigma_{yy}$.

At low collagen stiffness $k_{1}=10\;MPa$, there is a transition in the stress concentration at the interface during equi-biaxial loading illustrated in Fig. 4. At small deformation, the valleys on the side of the dermis interface (greater epidermis thickness) have a small stress compared to the epidermis in both $\sigma_{xx}$, $\sigma_{yy}$. This is in contrast to the results of Fig. 2. As deformation increases, the behavior qualitatively changes and the location of maximum stresses become the valleys of the dermis (greater epidermis thickness), similar to the results in Fig. 2. Fig. 4a shows that at λ=1.25 there is a transition in the distribution of $\sigma_{xx}$ across the interface. Beyond the λ=1.25 stretch, the patterns of Fig. 2 are recovered and the bands of high stress in the valleys of the dermis appear. These results suggest that even when loaded in the direction of anisotropy, epidermis mechanics contribute to and can dominate the overall tissue mechanical behavior at small deformations.

### The amplitude of the sinusoidal interface affects the stress concentration but not the homogenized response

**Fig. 5:**
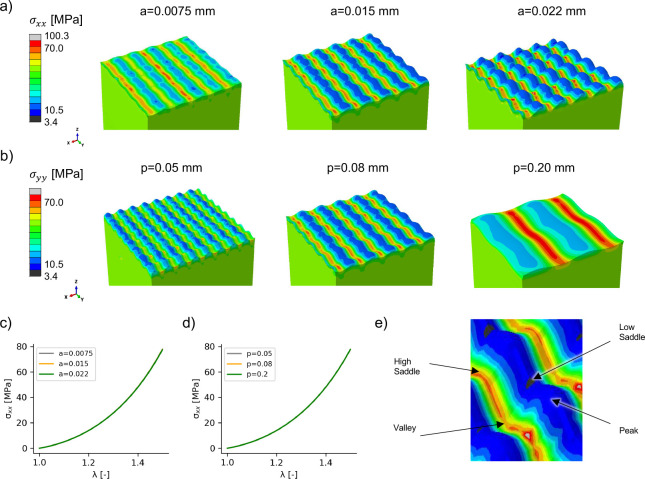
Amplitude but not period variations change the magnitude of the stress concentration. Under biaxial deformation, amplitude increases lead to higher and lower stresses at key interface locations (a), whereas the period redistributes the stress but without change in the magnitude of the stress concentration (b). The homogenized response is unchanged by either amplitude (c) of period (d) variations as expected. Higher stresses occur at the valley and high saddle compared to the peak and load saddle points (e).

The parameters of the sinusoidal interface were determined from analyzing histology images (see Supplemental material). However, even though we used the average values, variation in the parameters was observed. Further, variability between subjects and anatomical location is likely to contribute to even more variation on the Rete ridge geometry, which we captured as a sinusoidal interface. We thus tested different values of the amplitude and period of the parametric equation (eq. (1)). The period variation had an expected role, distributing the stress differently over the interface but not changing the values of the stress at the interface. In other words, the period changes are simply corresponding to different RVE dimensions but do not change the mechanics at the interface, see Fig. 5b.

The amplitude changes, on the other hand, changed the values of the stress at the interface, see Fig. 5a. The results are further documented in Table 4. In summary, the stress increases at the high saddle on the dermis side as the amplitude of the sine wave increased, and the stress decreased on the low saddle as the amplitude increased. Despite changes in the stress concentration, the homogenized response is unaffected by changes in the interface geometry, which was expected based on the results of Fig. 2.

### The stress jump is lower across a sinusoidal interface loaded in shear compared to the flat interface

**Fig. 6:**
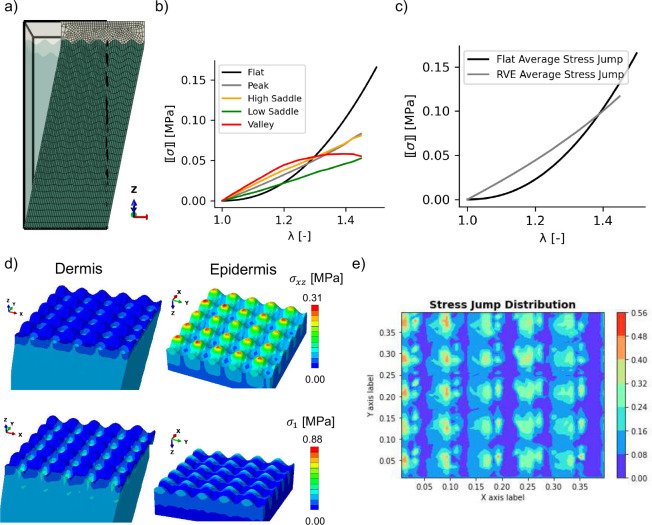
RVE with sinusoidal interface loaded in shear. a) Initial and final deformation of the RVE with shear deformation imposed by displacing the top surface in the x direction. b) Plots of the stress jump at different locations in the sinusoidal interface compared to the flat model, as well as c) the average stress jump in the model with the sinusoidal interface compared to the flat interface model. d) Contour plots for $\sigma_{xz}$ and maximum principal stress $\sigma_{1}$ show the expected stress concentrations on the dermis and epidermis sides of the interface. e) The contour of stress jump for the maximum principal stress $\sigma_{1}$ at the interface between epidermis and dermis.

The final simulations with the RVE depicted in Fig. 1b were the shear simulations in the x- and y-directions. Only the results for shear in x are shown in Fig. 6, the other shear results are reserved for the Supplemental Material. We were interested in the shear stress in the plane of deformation as well as the maximum principal stresses. Regions of stress concentration remained similar to previous observations under biaxial loading. The valleys of the dermis had higher stresses.

The jump in principal stress across the interface is plotted in Fig. 6. For the flat interface, the jump is computed based on the difference between the maximum principal stresses, which are aligned with $\sigma_{xx}$. As the model is loaded in shear, the jump in $\sigma_{xx}$ across the flat interface increases nonlinearly. In contrast, the stress jump across different key regions in the interface all show less magnitude compared to the flat interface at larger stretches. On average, the stress jump across the sinusoidal interface in shear was only greater than the flat interface at small deformation. As the deformation increased, the flat interface showed a much greater stress jump compared to the RVE model with the sinusoidal interface. Together with the biaxial deformation results, the shear simulations also support the role of the sinusoidal interface in reducing the stress jump across the epidermis and dermis in a nonlinear fashion as a function of deformation.

### Hair follicles induce strain concentration at the bulge and bulb regions

Under equi-biaxial loading, the RVEs with the hair follicle showed that the largest strains are concentrated around the hair follicle. For a notation of the different regions of the hair follicle please refer to the Supplementary figure. The bottom of the hair follicle is called the bulb. It is a hollow spherical region of epithelial tissue surrounded by the dermis. The bulb region shows increasing strains as a function of equi-biaxial deformation, with a clear region of high strain at stretches ≥ 1.18. The strains in the sinusoidal interface are low compared to the strains in the epithelial tissue surrounding the hair follicle. Interestingly, as the skin is biaxially stretched and the thickness reduced, the hair follicle shows a slight relative displacement with respect to the skin surface because the hair follicle is much stiffer and oriented perpendicular to the stretching of the skin.

Shear simulations showed an even more prominent role for the hair follicle. Even though the $\sigma_{xz}$ stresses do not show a particular spatial distribution, the principal strain reveals two regions of strain concentration. Once again, the two regions of high strain were located in distinct anatomical regions, the bulb at the base and the bulge region near the epidermis-dermis interface adjacent to the hair follicle. For the bulge, the strain concentration is at the transition between the spherical region and the epithelium covering the shaft of the hair, whereas the strain concentration near the bulge region occurred toward the opposite side.

## Discussion

In this study we showed that the sinusoidal epidermal-dermal interface contributes to the concentration of stress between both layers, which could have an impact on skin mechanophysiology. We observed that the homogenized response was independent of the interface geometry, but that this interface shape led to stress and strain concentrations at particular locations. Even though it increased the stress concentrations, the sinusoidal interface led to smaller stress jumps across the interface under both biaxial and shear loading. The biomechanics of the model with the hair follicle showed even more intricate distribution of the stress and deformation of the skin RVE. In particular, deformation was localized to the region of the epithelium surrounding the hair follicle right below the epidermis layer. The results from this study therefore add to our understanding of skin mechanophysiology and suggest the need for models that include skin heterogeneities in particular for studies interested in the delamination properties of the skin, for studies related to differential biological response driven by strain/stress concentrations such as tissue expansion, and studies interested in the sense of touch and deformation of skin mechanoreceptors embedded in particular sub-structures of this complex and heterogeneous tissue.

The role of rough interfaces in the delamination behavior of composites has produced knowledge that is the basis for understanding the results observed here in the context of skin biomechanics. Zavatieri et al. investigated crack propagation for sinusoidal interfaces across linear elastic materials [53]. In particular, they showed that for a sinusoidal interface of amplitude A and period λ, the stress intensity factor for mode-I crack propagation increased linearly with the ratio A/λ. In other words, the stress intensity factor, or effective fracture toughness, increased as the angle of rotation along the interface became sharper, in agreement with analytical models of mode-I fracture for a flat crack that suddenly needs to turn by an angle α [10]. Finite element simulations of fracture across other kinds of non-flat interfaces showed similar results [16]. Even though we did not perform fracture simulations, these earlier works point toward sinusoidal interfaces in biological tissues as a mechanism to increase effective fracture toughness against delamination. Indeed, it has been hypothesized that flattening of Rete ridges with ageing is one of the causes for increased risk of epidermis delamination in the elderly [26, 42]. There are several studies suggesting that the origin of Rete ridges is due to different growth rates of dermis and epidermis during development causing buckling and resulting in the wavy interface [37, 9]. However, unlike in other tissues, the role of buckling during development of skin was not tied to a mechanophysiological role in these studies. In contrast, for other tissues with similar wavy interface such as the brain or the gut, the obvious benefit of buckling is increased surface area [4, 47]. For the skin, a direct mechanical role of Rete ridges seems a logical hypothesis [6]. In support of this hypothesis, our simulations show that the stress jump across the interface decreases with the introduction of the sinusoidal interface. If the interface between dermis and epidermis is in fact the weakest material and the first to fracture under mechanical loading as suggested in the literature [50, 56], then reducing the stress jump across this interface and increasing its surface area is likely to optimize the energy dissipation of this failure process. Nevertheless, further work is needed to characterize the delamination properties of skin taking into consideration the nonlinear behavior of the different layers.

Computational models taking into consideration the different layers of skin have been developed before [11, 40, 30, 54, 12]. However, these efforts were focused on flat interface geometries between epidermis and dermis. We confirm that the sinusoidal interface is not needed if the emphasis is on the macroscale mechanical behavior because the homogenized response of the RVEs is independent of the interface geometry (Figs. 2, 5). In fact, the rule of mixtures and semi-analytical approaches from Fig. 1d,e are already appropriate for the homogenized response given that the macroscale behavior is dictated by the dermis, particularly under larger deformations, as is usually argued in models of the skin as a homogeneous material [29, 23, 34]. Multi-layer models are necessary for particular applications such as drug delivery or skin tribology [11, 39]. Here we show that the addition of the sinusoidal interface leads to specific patterns of stress/strain concentration which, beyond implications for fracture, might play a role in skin mechanobiology. At larger deformations, the valleys of the Rete ridges show the larger stresses. Interestingly, there are two conflicting hypothesis regarding the distribution of stem cells of the epidermis with respect to the Rete ridges, with evidence for two distinct stem cell populations, one at the peak of the Rete ridges [22, 19], and one at the valleys [41, 48]. It remains unclear if the mechanics or the geometry alone of the interface have a distinct role in the preferential accumulation of stem cells in these regions, but it is clear that these two regions have distinct states of stresses under tensile loading. Investigating the mechanobiology of stem cell sub-populations is a natural direction for future research. It has also been established that loss of the stem cell populations with ageing results in the flattening of the Rete ridges and contributes to skin fragility [19]. A key insight from our simulations is that the resulting stress concentrations are three-dimensional in nature. While most analysis of Rete ridges in either experiments or simulations simplify the skin to a two-dimensional body under plane strain, we show that the biomechanics of the interface are inherently three-dimensional. For instance, we show that skin anisotropy can lead to bands of higher stress at the interface that extend across valleys and saddle points of Rete ridges (Fig. 2). Rather than two, we suggest to consider four locations to fully characterize the state of stress at the interface, the peaks and valleys but also the saddle points (Fig. 5).

Even though skin appendages are a ubiquitous feature of histology and key for skin mechanophysiology, e.g., sense of touch, they are completely absent in skin biomechanics studies. We included a hair follicle in our simulation to determine the strain distribution in the presence of this particular heterogeneity. Our emphasis on strain concentrations induced by hair follicles originated from the known roles of hair in mechanosensation [17]. There are five types of mechanoreceptors in hairy skin in many mammals such as mice and humans [25]. Their classification depends on the conduction velocity of the action potential from the receptor to the spinal cord, as well as their capacity to adapt to sustained loading [21]. We showed that under equi-biaxial deformations the strains concentrate first at the epidermis region immediately adjacent to the hair follicle. At increasing deformation, more of the epithelium surrounding the hair become stretched. The epidermis region adjacent to hair follicles is characterized by the presence of Merkel cells, which are prominent mechanoreceptors [25, 51]. The epithelium surrounding the hair follicle itself, just below the epidermis, contains different kinds of slow and fast acting mechanoreceptors. What is most interesting is that these receptors come in two different type of nerve ending alignment, either circumferential around the hair follicle [3], or in lanceloate endings aligned with the axis of the hair follicle [28]. The fact that maximum strains under physiological loading coincide with distinct location of mechanoreceptors in the skin suggests a possible optimization of these receptors to particular type of deformations. The concentration of strain is even cleared under shear, for which high strains exist near the bulge region of the hair follicle, where the circumferential and lanceloate nerve endings are known to exist [3, 28]. However, the bulb region of the hair follicle also showed strain concentrations, particularly under shear, but this region is not a primary locus of mechanoreceptors [21]. Future work should investigate the strain patterns achieved by specific type of deformations beyond the ones studied here.

## Conclusion

Previous work has studied multilayered models of skin but little effort has been invested in understanding the role of the epidermal-dermal interface and how it affects the overall skin mechanics. Here, we found that the overall stress-strain response is not affected by the geometry of the interface but that there were changes in local stresses at the epidermal-dermal interface. Stresses increased with increasing amplitude of the Rete ridges, particularly at the high saddle points of the interface. The stress at this location was about 62 MPa (at a stretch of 1.3) for an amplitude of 0.0075 mm and 77 MPa for an amplitude of 0.022 mm. Even though the presence of Rete ridges increased stress at some locations, the total interface surface was larger and the average stress jump decreased with the presence of the sinusoidal interface, which can explain a physiological role of this interface geometry in preventing delamination. There was also clear evidence that heterogeneities, such as hair follicles, disrupt the mechanics of skin, showing that these heterogeneities lead to stress/strain concentration in the epithelium and the epidermal-dermal interface where mechanorecepters are located. This might have an influence on skin mechaniobiology as different touch-sensin cells are exposed to differential stresses and strains.

## Figures and Tables

**Fig. 7:**
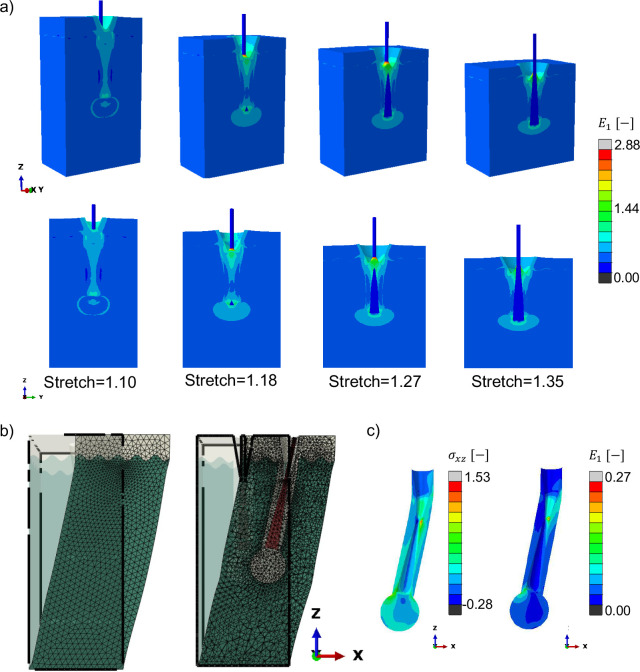
RVE with hair follicle under equi-biaxial and shear loading. a) Under equi-biaxial loading, maximum principal strain $E_{1}$ concentrates in the epithelium surrounding the hair follicle due to the contrast in stiffness between the dermis, epithelium, and hair follicle materials, with the epithelial tissue the softest of the three. In particular, strains concentrate at the bulb of the hair follicle and at the epidermis level. b) Under shear, even though the $\sigma_{xz}$ stresses are distributed in the entire epithelium around the hair follicle (c), strains $E_{1}$ are localized at the bulb and the bulge regions (d).

**Table 1:** Ogden material parameters for the epidermis [15]

$\mu_{E}$	α	$D_{E}$
6.1105 MPa	2.9814	0.0164 MPa^−1^

**Table 2:** Gasser-Ogden-Holzapfel material parameters for dermis [36]

$\mu_{D}$	$k_{1}$	$k_{2}$	k	$D_{D}$
0.2014 MPa	24.53 MPa	0.1327 MPa	0.14	0 MPa^−1^

**Table 3:** Neo-hookean material parameters for the hair follicle [18]

$\mu_{G}$	$D_{H}$
1.57 GPa	0.0003 MPa^−1^

**Table 4:** Stress values at various locations along the interface for amplitude variation a in mm. Stresses are reported in MPa and the quantity in parenthesis is the standard deviation (SD).

Location	a = 0.0075	a = 0.015	a = 0.022
Valley	61.02 (4.05)	62.34 (0.41)	60.22 (0.32)
High Saddle	61.38 (4.44)	66.56 (1.21)	72.80 (0.94)
Peak	20.53 (1.56)	14.11 (0.20)	14.50 (0.23)
Low Saddle	21.87 (1.68)	13.66 (0.22)	10.69 (0.25)
